# Designing High Interfacial Conduction beyond Bulk via Engineering the Semiconductor–Ionic Heterostructure CeO_2−δ_/BaZr_0.8_Y_0.2_O_3_ for Superior Proton Conductive Fuel Cell and Water Electrolysis Applications

**DOI:** 10.1021/acsaem.2c02995

**Published:** 2022-12-15

**Authors:** Yueming Xing, Bin Zhu, Liang Hong, Chen Xia, Baoyuan Wang, Yan Wu, Hongdong Cai, Sajid Rauf, Jianbing Huang, Muhammad Imran Asghar, Yang Yang, Wen-Feng Lin

**Affiliations:** † Engineering Research Center of Nano-Geo Materials of Ministry of Education, Faculty of Materials Science and Chemistry, China University of Geosciences, No. 388 Lumo Road, Wuhan430074, China; ‡ Jiangsu Provincial Key Laboratory of Solar Energy Science and Technology/ Energy Storage Joint Research Center, School of Energy & Environment, Southeast University, Nanjing210096, China; § Department of Chemical Engineering, Loughborough University, Loughborough, LeicestershireLE11 3TU, U.K.; ∥ Hubei Collaborative Innovation Center for Advanced Organic Materials, Faculty of Physics and Electronic Science, Hubei University, Wuhan430062, China; ⊥ College of Electronics and Information Engineering, Shenzhen University, Nanshan, Guangdong Province518000, China; # State Key Laboratory of Multiphase Flow in Power Engineering, Xi’an Jiaotong University, Xi’an710049, China; ∇ New Energy Technologies Group, Department of Applied Physics, Aalto University School of Science, P. O. Box 15100, Aalto, FI-00076Espoo, Finland

**Keywords:** ceramic proton-conducting electrolyte, proton ceramic fuel cells, solid oxide water electrolysis cell, semiconductor−ionic heterostructure, interface engineering

## Abstract

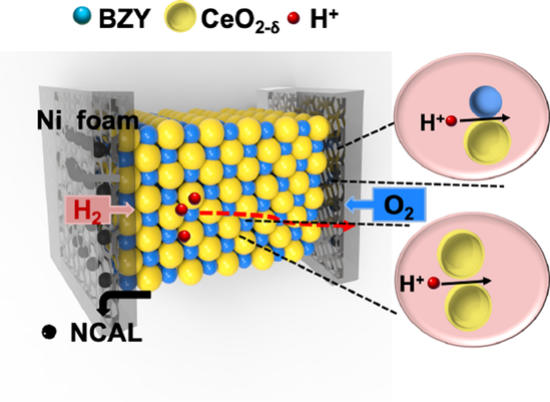

Proton ceramic fuel cells (PCFCs) are an emerging clean energy technology; however, a key challenge persists in improving the electrolyte proton conductivity, e.g., around 10^–3^–10^–2^ S cm^–1^ at 600 °C for the well-known BaZr_0.8_Y_0.2_O_3_ (BZY), that is far below the required 0.1 S cm^–1^. Herein, we report an approach for tuning BZY from low bulk to high interfacial conduction by introducing a semiconductor CeO_2−δ_ forming a semiconductor–ionic heterostructure CeO_2−δ_/BZY. The interfacial conduction was identified by a significantly higher conductivity obtained from the BZY grain boundary than that of the bulk and a further improvement from the CeO_2−δ_/BZY which achieved a remarkably high proton conductivity of 0.23 S cm^–1^. This enabled a high peak power of 845 mW cm^–2^ at 520 °C from a PCFC using the CeO_2−δ_/BZY as the electrolyte, in strong contrast to the BZY bulk conduction electrolyte with only 229 mW cm^–2^. Furthermore, the CeO_2−δ_/BZY fuel cell was operated under water electrolysis mode, exhibiting a very high current density output of 3.2 A cm^–2^ corresponding to a high H_2_ production rate, under 2.0 V at 520 °C. The band structure and a built-in-field-assisted proton transport mechanism have been proposed and explained. This work demonstrates an efficient way of tuning the electrolyte from low bulk to high interfacial proton conduction to attain sufficient conductivity required for PCFCs, electrolyzers, and other advanced electrochemical energy technologies.

## 1 Introduction

High ionic transport properties are the central focus of solid-state energy devices, especially in proton ceramic fuel cells (PCFCs), which is an emerging trend for solid oxide fuel cell research and development.^1,2^ However, the conventional proton conducting ceramic electrolyte, yttrium-doped barium zirconate (BZY) perovskite, shows a typical ionic conductivity of ca. 10^–3^–10^–2^ S cm^–1^ at 600 °C, which is one to two orders of magnitude lower than the required threshold of 0.1 S cm^–1^ that is offered by the commercial Nafion proton-exchange membrane electrolyte. Furthermore, the preparation of the BZY requires a high-temperature (>1300 °C) sintering densification process.^3^ In order to improve the ionic conductivity of the BZY, which is based on the bulk conducting mechanism, a high-temperature sintering process could be applied to reduce the detrimental effect of the grain boundaries on ionic transport. Moreover, some structural doping strategies have been employed, including doping transition metal elements, e.g., BaZr_0.9–*x*_Fe_*x*_Y_0.1_O_3−δ_,^4^ BaCo_0.4_Fe_0.4_Zr_0.1_Y_0.1_O_3−δ_ (BCFZY_0.1_) and Ni-doping in BCFZY (Ba(Co_0.4_Fe_0.4_Zr_0.1_Y_0.1_)_0.95_Ni_0.05_O_3−δ_)^5,6^ and rare earth metal elements, such as Ce^4+^ at the B site of the perovskite structure to enhance the proton conductivity in the perovskite materials, and BaZr_0.1_Ce_0.7_Y_0.2_O_3−δ_,^7^ but the ionic conduction is still hindered, e.g., the conductivity at 600 °C is far below the benchmark 0.1 S cm^–1^. Therefore, it is a big challenge to improve the ionic conductivity of the protonic ceramic materials through the structural design and ion-doping.

Semiconductor-ion materials (SIMs) have become promising candidates to replace traditional ionic electrolyte materials.^8−11^ The discovery of SrTiO_3_-YSZ is a typical example of SIMs.^12^ Although the ionic conductivity of the interfaces has been reported to increase by eight orders of magnitude as compared to the YSZ, its applications for fuel cells have not been reported yet, perhaps due to the electronic conduction of SrTiO_3_. Chen et al. reported that the semiconductor La_0.25_Sr_0.75_TiO_3_ (LST) exhibited a surface superionic conduction by constructing a self-heterostructure.^13^ Zhu et al. reported the La_0.6_Sr_0.4_Co_0.2_Fe_0.8_O_3−δ_ (LSCF) and Sm–Ca co-doped ceria (SCDC) heterostructure material.^14^ The synergistic effect of electronic and ionic conductions of the junction is aligned toward the level of charge separation, especially preventing electron crossover, and at the same time leading to an enhanced ionic conductivity. Such a semiconductor–ionic heterostructure (SIH) promotes ion transport up to 0.1 S cm^–1^ conductivity in a temperature range of 500∼550 °C and yields a synergic ionic transport and much-enhanced fuel cell power density, compared to that of the pure ionic SCDC electrolyte fuel cell. The deep scientific understanding of the mechanism behind such an SIH system requires further investigation. Yousaf et al. reported another SIH system, the Ni_0.4_Zn_0.6_Fe_2_O_4_ (Ni–Zn ferrite) and Sm_0.2_Ce_0.8_O_2_ (SDC),^15^ by replacing the ionic SDC electrolyte, which exhibited significant effects on improving the fuel cell performance, i.e., from 615 mW cm^–2^ using the SDC electrolyte to 760 mW cm^–2^ using the SIH at 550 °C. Mushtaq et al. built a SIH by using a more stable semiconductor, SrFe_0.75_Ti_0.25_O_3−δ_ (SFT) and SDC.^16^ It exhibited a high ionic conductivity >0.1 S cm^–1^ at 520 °C, which is an order of magnitude higher than that of the SDC.

Many studies have demonstrated that the interface plays a key role in the functions of the device, including both the semiconductor–ionic and the semiconductor–semiconductor interfaces. For example, Xia et al. developed nanocomposite hematite–LaCePrOx (hematite–LCP) as an electrolyte candidate exhibiting a high conductivity of 0.116 S cm^–1^ at 600 °C due to its heterophasic interface.^17^ The emergence of SIH or heterostructure materials, in general, can effectively enable the interface to provide excellent electrical properties and device performances.^18,19^ Zhu et al. summarized that the semiconductor and heterostructure materials carried much higher ionic conductivities than those of single-phase oxide ion conducting electrolytes at low temperatures.^20^ Rauf et al. designed triple-charge conducting semiconductor oxide based on Ba_0.5_S_r0.5_Co_0.1_Fe_0.7_Zr_0.1_Y_0.1_O_3−δ_ (BSCFZY) which formed a heterostructure with Ca_0.04_Ce_0.80_Sm_0.16_O_2−δ_ (SCDC).^21^ It is demonstrated that the formation of a heterointerface between BSCFZY and SCDC helps in providing high ionic conductivities. Wang et al. reported that the 3% (by mol) Ni-doped surface in Sm_2_O_3_ builds up continuous surfaces as proton channels for high-speed transport as an electrolyte for fuel cells.^22^ Furthermore, Akbar et al. reported a perovskite semiconductor BaSnO_3_ (BSO), in which oxygen vacancies play a vital role in promoting proton transport.^23^ As for the proton diffusion mechanisms, Shao et al. reported and summarized three mechanisms of the proton conduction.^24^ The first is the Grotthuss mechanism, where H^+^ diffuses through a network of bond breaking and hydrogen bonding of water molecules. The second is the vehicle mechanism, where H^+^ transports through the formation of OH radicals and oxygen vacancies (*V*_o_) (e.g., perovskite type). The third is the interfacial conduction, which is more efficient. Due to the formation of oxygen vacancies and the unbalanced charge distribution of the metal vacancies, a local electric field (LEF) was formed at the heterojunction interface. The LEF promotes H^+^ diffusion and migration along with the interface. Therefore, it is expected that the fast proton transport promoted by the semiconductor heterostructure field could make the operating temperature of ceramic fuel cells down to 500° and even to 300 °C.^24,25^ Therefore, in the relatively low temperature working conditions, the interface conduction, which is superior to the bulk conduction, should be developed as a new methodology for fabricating high-performance proton conductors for advanced PCFCs. CeO_2_ exists as a chemistry deficit form CeO_2−δ_, due to Ce^4+^/Ce^3+^ redox being easily occurring at the surface, which made ceria a strong semiconductor with a direct bandgap (∼3.2 eV). Asghar et al. suggested that the wide bandgap semiconductors can be used for the electrolytes in fuel cells.^26^

In this work, we have developed a new methodology to tune BZY from a low bulk proton conductivity to a high interfacial conductivity through the SIH. Beyond the bulk conducting mechanism, we first demonstrated that proton conduction can be increased by the grain boundary, resulting in enhanced conductivity and PCFC power output; subsequently based on the new interface conducting mechanism, we further constructed the heterostructures between BZY and CeO_2−δ_. A record-high proton conductivity up to 0.23 S cm^–1^ at 520 °C has been achieved for the CeO_2−δ_/BZY SIH through the interfacial conduction. Furthermore, excellent PCFC performances have been demonstrated, with a high power density of 845 mW cm^–2^ obtained from the CeO_2−δ_/BZY SIH electrolyte in comparison to 229 mW cm^–2^ from the BZY electrolyte.

**Figure 1:**
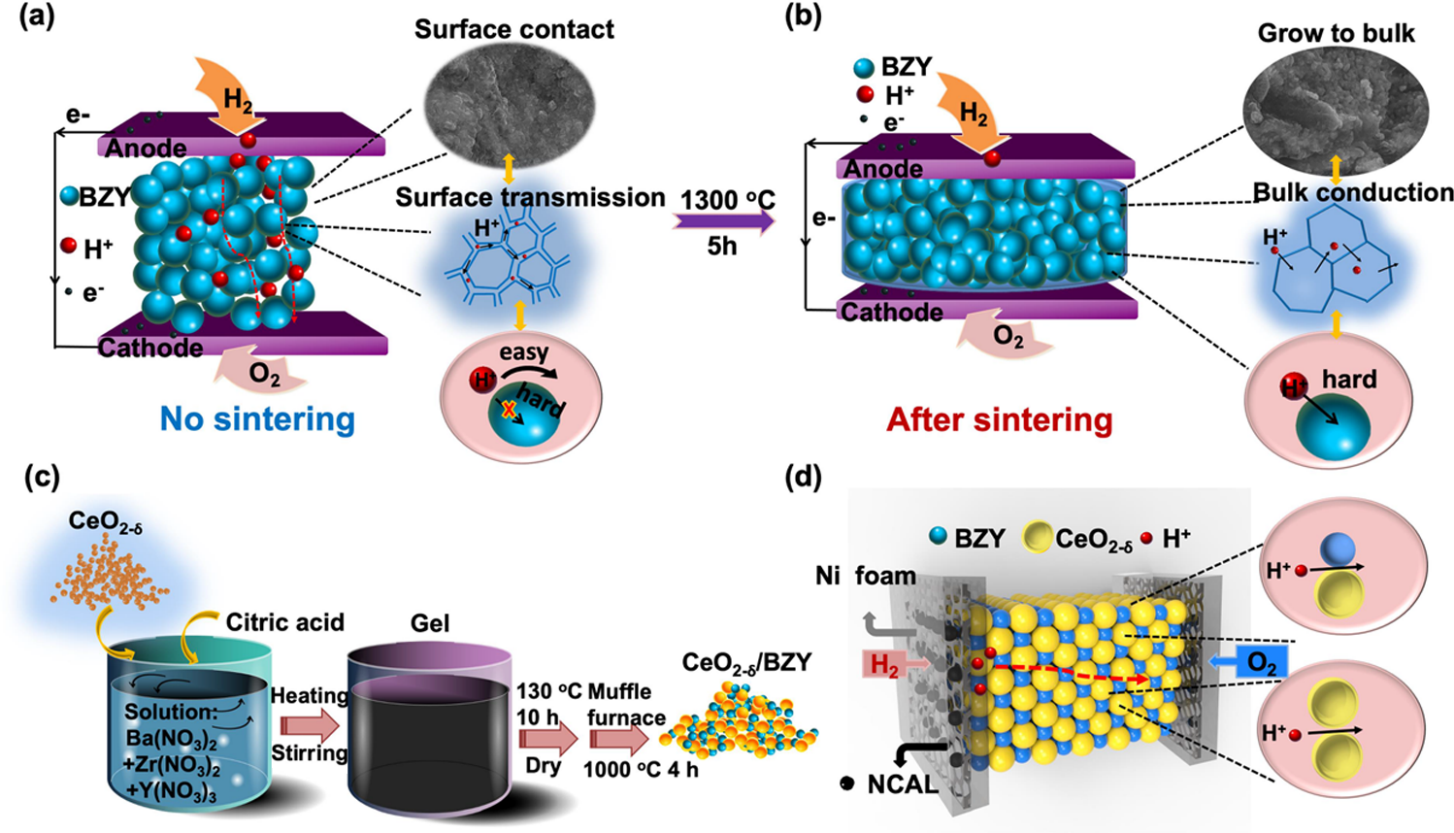
Schematic of the proposed design ideas for surface proton conduction enhancement, where (a) BZY without sintering was used as the electrolyte layer; (b) BZY was sintered after high-temperature treatment; (c) synthesis process of CeO_2−δ_/BZY; (d) fuel cell assembly using Ni-NCAL/(CeO_2−δ_/BZY)/NCAL-Ni as the anode/electrolyte/cathode.

Figure 1 presents the new methodology we developed to tune the bulk to grain boundary conduction for BZY as illustrated in Figure 1a and further to interfacial conduction in the CeO_2−δ_/BZY SIH. We employed the triple conducting semiconductor Ni_0.8_Co_0.15_Al_0.05_LiO_2_ (NCAL) symmetric electrodes and BZY as the electrolyte in a configuration of the Ni foam-NCAL/BZY/NCAL-Ni foam fuel cell device. Figure 1a presents the BZY electrolyte layer without being subjected to the high-temperature sintering, where the particles connected with each other to form a spider-like grain boundary, which can promote proton transport beyond the crystal structure so that the grain boundary (particle surface) conduction is the major and optimal path for proton conductivity. For comparison, the BZY layer was subjected to a high-temperature sintering process to eliminate the grain boundary effect as shown in Figure 1b. The BZY particles are densified, leading to only the structural bulk conduction pathways. Based on this approach, we further developed a BZY coating on CeO_2−δ_ to form a CeO_2−δ_/BZY SIH; the synthesis process is presented in Figure 1c. The prepared CeO_2−δ_/BZY replaced the BZY electrolyte layer in the fuel cell with the configuration of a layered Ni-NCAL/(CeO_2−δ_/BZY)/NCAL-Ni (see Figure 1d). In this case, the ion transport was further increased with more paths available; in addition, the built-in electric field (BIEF) formed in the CeO_2−δ_/BZY SIH promoted the proton transport through the interfaces. More importantly, this significant improvement in the proton transport could enable the fuel cell to be operated in the reversed electrolysis mode where a high current density would be desired for water electrolysis to produce hydrogen and oxygen at cathode and anode sides, respectively.

## 2 Experimental Section

### 2.1 Synthesis of Materials and Fabrication of Fuel Cells

BZY: 0.01 mol of Ba(NO_3_)_2_ (99.9%, Wako), Zr(NO_3_)_3_ (97%, Wako), and Y(NO_3_)_3_ (99.8%, Aldrich) were dissolved in 300 mL of deionized water. Then citric acid monohydrate solution was added into the above mixed nitrate solution drop-by-drop. The mixture was continually stirred at 120 °C until being evaporated to gel, which was dried at 130 °C overnight. The obtained powder was placed into a muffle furnace for sintering at 300 °C for 1 h, then at 1300 °C for 5 h, with a heating rate of 5 °C/min, to obtain BZY powder.

CeO_2−δ_: 0.01 mol of Ce(NO_3_)_3_ and 0.09 mol Na_2_CO_3_ were dissolved in 250 mL and 150 mL of deionized water, respectively. Then the Na_2_CO_3_ solution was added to Ce(NO_3_)_3_ solution slowly under stirring. The mixed solution was stirred continuously for 5 h, resulting in a white precipitate. The precipitate was filtered and washed with deionized water several times until the solution was neutral, and then it was dried at 80 °C in an oven for 24 h. The obtained white power was heated at 700 °C for 2 h in a muffle furnace, resulting in a light-yellow powder named CeO_2−δ_.

CeO_2−δ_/BZY: 0.020 mol Ba(NO_3_)_2_, 0.016 mol Zr(NO_3_)_3_, and 0.004 mol Y(NO_3_)_3_ were dissolved in 300 mL of deionized water, then 0.100 mol CeO_2−δ_ powder was added into the solution carefully under stirring, and the stirring was maintained for 4 h. The citric acid solution was then dripped into the above solution under stirring at 120 °C to allow the evaporation of the water to reach the dryness of the precipitate. Finally, the precipitated heterostructure material of CeO_2−δ_/BZY was collected, and it has a density of 6.25 g cm^–2^ and a volume ratio of 2.73:1 (CeO_2−δ_:BZY). For fuel cell fabrication, 0.35 g of BZY powder was pressed with a pressure of 225 MPa to form a pellet of 13 mm diameter. The obtained pellet of 0.89 mm thickness was used as the electrolyte and was sandwiched between two NCAL (Ni_0.5_-Co_0.45_Al_0.05_LiO_2_) pasted on Ni foams as the electrodes; the resulting Ni-NCAL/BZY/NCAL-Ni assembly was used for fuel cell testing. Another BZY pellet was heat-treated at 1300 °C for 5 h to prepare the sintered BZY, which has a thickness of 0.76 mm and a density of 7.19 g cm^–2^. The 0.300 g CeO_2−δ_/BZY powder was taken each time and pressed in the same way to make the pellets, two of which were subjected to the sintering at 600 and 1000 °C, respectively, to form the sintered CeO_2−δ_/BZY-600 (6.66 g cm^–2^) and CeO_2−δ_/BZY-1000 (7.29 g cm^–2^) pellets.

### 2.2 Material Characterization

The phase structure was detected by X-ray diffraction (XRD) using a Bruker D8 (Germany, Bruker corporation) diffractometer with a Cu Ka radiation source over the 2θ range of 20–90°. Transmission electron microscopy (TEM) images and high-resolution transmission electron microscopy (HR-TEM) images of the samples were investigated by a Philips CM12/STEM Transmission Electron Microscope with an accelerating voltage of 120 kV. JSM7100F made of Japan was used to examine the morphology of the samples. Additionally, X-ray photoelectron spectroscopy (XPS) was performed with an ESCALAB 250Xi photoelectron spectrometer (Thermo Fisher Scientific, UK). The optical energy bandgap of the materials was estimated by UV–vis–NIR absorption carried out on UV3600. Ultraviolet photoelectron spectroscopy (UPS) analysis was carried out using an X-ray photoelectron spectrometer instrument (AXIS-ULTRA DLD-600 W, Shimadzu Japan) with He I radiation (21.2 eV). Kelvin probe force microscopy (KPFM, Bruker Multimode 8) was employed to detect the surface potential of the CeO_2−δ_/BZY heterostructure with tapping mode. The two-probe DC Hebb–Wagner polarization method was used to determine electronic contribution in the CeO_2−δ_-BZY heterostructure, in which a constant voltage was applied on the conductivity cell using a Keithley 2400 source meter in a cell configuration of Ag/CeO_2−δ_-BZY/Ag, and current vs time (*I*–*t*) data were recorded by applying a bias voltage of 1.0 V. When the *I*–*t* curve is stable, the resistance data were obtained by dividing the voltage by current, i.e., *R* = *V*/*I*, and the electronic conductivity was further calculated.

### 2.3 Electrochemical Measurements

The electrochemical impedance spectroscopy (EIS) measurements were carried out using a Zennium-E (ZAHNER, Germany) in a frequency range of 0.1–10^6^ Hz. A fuel cell tester (ITECH DC ELECTRONIC LOAD, IT8511) was used to test the performance of the fuel cells, over the temperature range from 430 to 520 °C, where hydrogen and air were supplied as the fuel and oxidant, respectively, with a flow rate range of 100–120 mL min^–1^ under 1 atm to each side of the fuel cell. The stability testing was performed at 520 °C with a current density of 100 mA cm^–2^. For the reversed fuel cell mode, i.e., water electrolysis, the temperature of feeding deionized water was automatically adjusted to 70 °C by an electrothermal temperature controller, so that the water vapor was supplied to the positive electrode side (now anode in the electrolysis mode but was cathode in the fuel cell mode) through a gas pipe wrapped with insulation cotton and a constant temperature belt and a steam partial pressure of 31.2 MPa. The current–voltage (*I–V*) polarization data were collected by a two-point DC polarization test, performed using a digital source meter (Keithley 2400).

## 3 Results and Discussion

**Figure 2:**
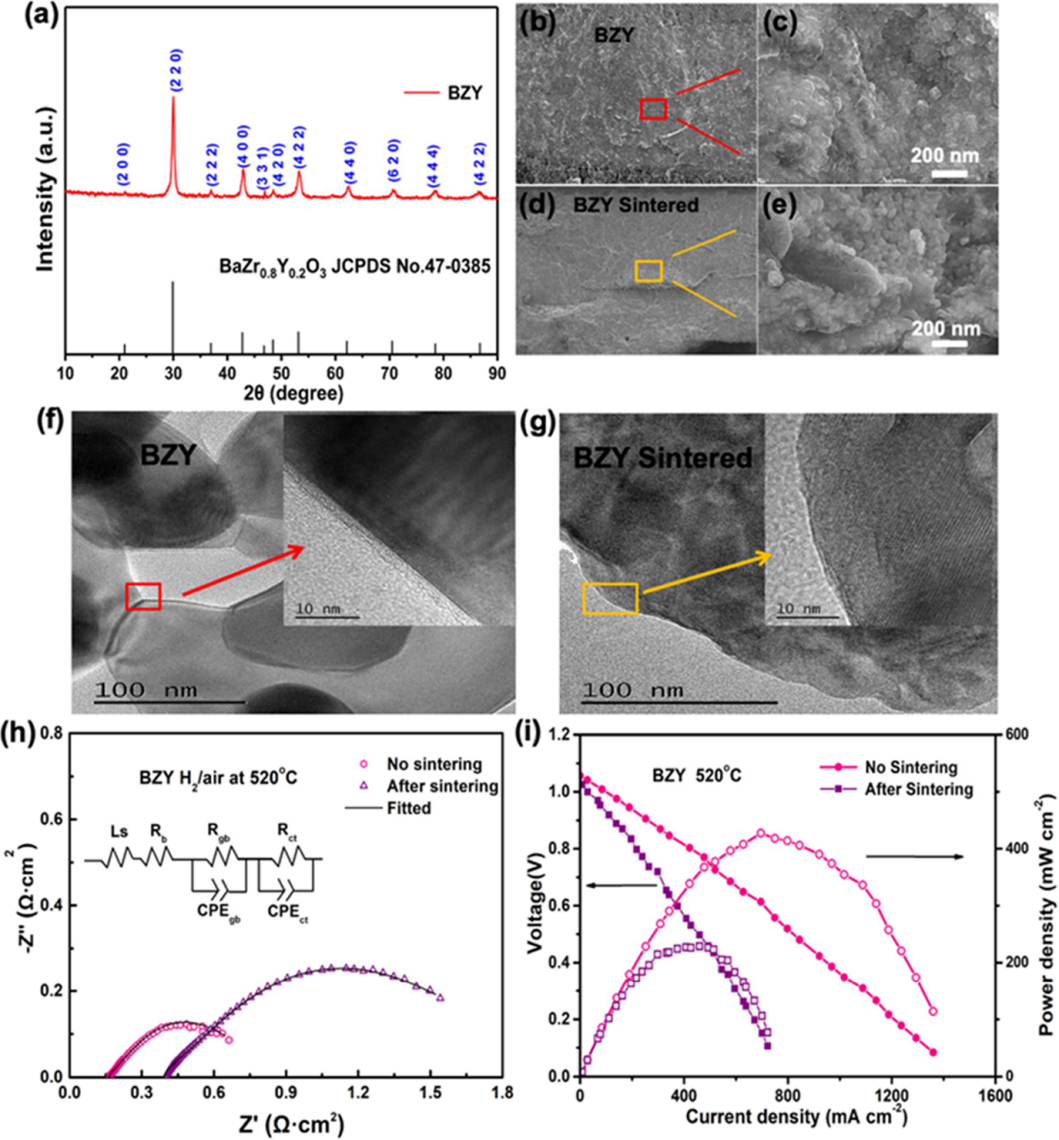
(a) XRD pattern of BZY; (b,c) cross-sectional SEM images of BZY; (d,e) cross-sectional of the BZY pellet after 5 h sintering at 1300 °C; (f,g) HR-TEM images of BZY without (f) and with (g) the sintering; (h) electrochemical impedance spectra and (i) *I*–*V* and *I*–*P* polarization data obtained from fuel cells using the BZY pellet electrolyte without and with the sintering.

The XRD pattern of BZY is shown in Figure 2a, corresponding to BaZr_0.8_Y_0.2_O_3_ (JCPDS No. 47–0385). The crystal planes of the main peaks are divided into (2 2 0), (4 0 0), (4 2 2), and (4 4 0), respectively. The 0.300 g BZY powder was pressed into a pellet with 13 mm in diameter which has 0.89 mm in thickness and a density of 6.14 g cm^–2^. The pellet was then sintered at 1300 °C for 5 h to make the dense ceramic membrane which has a thickness of 0.76 mm and a density of 7.19 g cm^–2^. It is obvious that after the high-temperature sintering, the thickness reduced while the density increased, and the particles were in tight contact and grew larger, which eliminated the boundary effect. The scanning electron microscopy (SEM) cross-sectional images of BZY before and after the sintering are shown in Figure 2b–e. After sintering, the particles have grown into connected large grains (comparing Figure 2c with e). Furthermore, TEM and HR-TEM were carried out, and the images are shown in Figure 2f,g. An obvious amorphous layer at the lattice boundary of BZY was observed, indicating that there are many surface defects. After the high-temperature sintering, the amorphous layer disappeared and a perfect lattice formed, indicating that the defects on the surface were removed. The sintering probably resulted in the perfect lattice structure which in turn blocked the ion transmission, because the contacts and grain boundaries among particles were significantly reduced, leading also to a large increase in the resistance, as shown in Figure 2h.

To probe the bulk and grain boundary effects, we assembled the fuel cell devices using the BZY pellets with and without sintering as the electrolyte, which was sandwiched between the two NCAL electrodes. The EIS curves obtained on BZY without and with sintering were compared as shown in Figure 2h, where the results were modeled through an equivalent circuit (inset) mode of *L*_s_*R*_b_(*R*_gb_CPE_gb_)(*R*_ct_CPE_ct_) as reported previously,^27,28^ where *R*_b_, *R*_gb,_ and *R*_ct_ stand for resistance of the bulk, the grain boundary, and the overall electrode, respectively. After high-temperature sintering of BZY, the resistance of the grain boundary increased from 0.59 to 2.95 Ω, also listed in Table S1 of the Supporting Information. This indicates that the grain boundary for the sample sintered at a high temperature is unfavorable for ion transport. Figure 2i shows the *I*–*V* and *I*–*P* polarization curves of the two fuel cell devices employing BZY without and with sintering treatment. The open-circuit voltages (OCVs) are 1.06 and 1.03 V for the fuel cell using BZY without and with sintering, respectively. The slightly lower OCV reflects some electronic short circuit phenomenon in the sintered BZY device. At 520 °C, the fuel cell power output reached 229 mW cm^–2^ for the sintered BZY electrolyte but 427 mW cm^–2^ for the BZY without sintering. The high-temperature sintering can decrease grain boundaries of the contacts among particles and tune the BZY conduction toward the bulk, while without high-temperature sintering, the BZY can maintain more grain boundaries/contacts to benefit ion transport leading to a higher fuel cell power output. This demonstrates that the grain boundaries can provide facile paths for proton transport with a much lower resistance compared to that from the bulk. Based on this mechanism, we further developed the interfacial proton conduction by constructing a SIH of CeO_2−δ_/BZY and then employed this as a new electrolyte for fuel cell and electrolysis cell operations.

**Figure 3:**
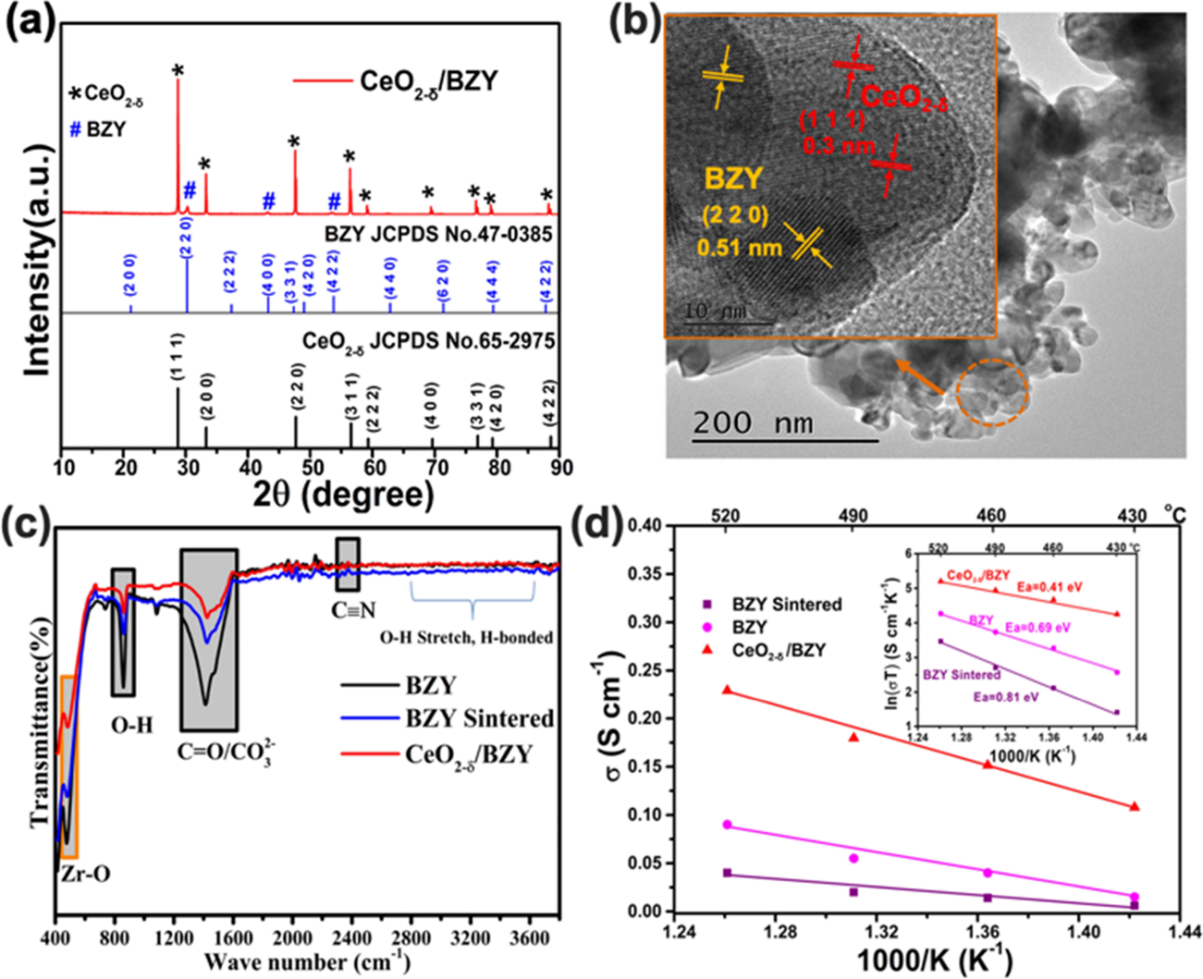
(a) XRD pattern of CeO_2−δ_/BZY; (b) TEM and HR-TEM images of the CeO_2−δ_/BZY heterostructure; (c) FT-IR spectra and (d) Arrhenius plots and calculated ion transport activation energies of BZY, BZY-sintered, and CeO_2−δ_/BZY.

The XRD pattern of CeO_2−δ_/BZY is shown in Figure 3. The diffraction peaks of the heterostructure material clearly show a two-phase composition, which corresponds to BZY and CeO_2−δ_, i.e., a heterostructure CeO_2−δ_/BZY composite. There is no other peak observed in the XRD pattern, eliminating the possibility of a new phase being formed. The elemental mapping of CeO_2−δ_/BZY was characterized using field emission SEM coupled with an energy-dispersive spectrometer (EDS), as shown in Figure S1. Figure S1a shows the SEM image of CeO_2−δ_/BZY particles, and Figure S1b–f shows the elemental mappings, showing that Ce, Ba, Zr, Y, and O are distributed uniformly in the sample. HR-TEM displays the heterojunction structure, see Figure 3b, where CeO_2−δ_ exposes mainly crystal plane (1 1 1), with a lattice spacing of 0.30 nm, while BZY exposes mainly (2 2 0) with a spacing of 0.51 nm; and clear boundaries and contacts between these two domains forming interfaces (highlighted by the circle in Figure 3b), i.e., the existence of two-phase heterojunction interfaces. Figure 3c shows the IR spectroscopy analysis of the BZY without and with sintering at 1300 °C, and the heterostructure of CeO_2−δ_/BZY. The IR spectra show clearly the functional groups contained in these materials, e.g., −OH stretching vibrations at the peaks around 2800–3500 cm^–1^ and 820 cm^–1^; C≡N stretch around 2338–2360 cm^–1^; and C=O/CO_3_^2–^ IR absorption around 1414–1428 cm^–1^, respectively.^29,30^ A peak around 506 cm^–1^ shows the formation of Zr–O. The IR peak intensity of C=O/CO_3_^2–^ decreases with the sintering of BZF and for the heterostructure of CeO_2−δ_/BZY. It is worth noting that the CeO_2_ peaks cannot be identified clearly in the heterostructure of CeO_2−δ_/BZY.^31^

Figure 3d presents the Arrhenius plots of BZY, BZY-sintered, and the heterostructure material CeO_2−δ_/BZY. The temperature-dependent conductivities of these three materials were obtained from the EIS testing results under fuel cell operating conditions. The electronic conductivity was obtained by the DC polarization method, where a 1.0 V bias voltage was applied to obtain the current/time curve as shown in Figure S2 of the Supporting Information, when the curve was stable, the resistance was obtained by dividing the voltage by the current, and then the electronic conductivity can be calculated. The ionic conductivity was obtained from eq 1:
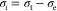
1where σ_*i*_ is the ionic conductivity, σ_t_ is total conductivity from EIS (more details are listed in Table S1 of the Supporting Information), and σ_e_ is electronic conductivity from DC testing (more details are shown in Figure S2 of the Supporting Information). The calculation results of the total conductivity, electronic conductivity, and ionic conductivity are listed in Table 1. The measured electronic conductivity is very low and can be negligible, and the ionic conductivity is approximately equal to the total conductivity.

**Table 1:** Lists of Key Performance Data Obtained from BZY-Sintered, BZY, and CeO_2−δ_/BZY at 520 °C

	*E*_a_	LnA	*A*	*C*	σ_t_/S cm^–1^	σ_e_/S cm^–1^	σ_*i*_/S cm^–1^
BZY-sintered	0.81	3.69	4.01E+01	4.01E+01•k	0.04	0.00129	0.039
BZY	0.69	4.49	8.92E+01	8.92E+01•k	0.09	6.16E–08	0.09
CeO_2−δ_/BZY	0.41	5.34	2.09E+02	2.09E+02•k	0.23	1.17E–05	0.23

At 520 °C, as listed in Table 1 the ionic conductivities of BZY-sintered, BZY, and CeO_2−δ_/BZY were 0.039, 0.09, and 0.23 S cm^–1^, respectively. The corresponding ionic conducting activation energy decreased from 0.81 eV (BZY-sintered) to 0.69 eV (BZY) and 0.41 eV (CeO_2−δ_/BZY). These data show clearly that heterostructure CeO_2−δ_/BZY has the highest ionic conductivity among the three samples studied, while sintered BZY has the lowest one, suggesting that ion transport on the surface is significantly faster than that in the bulk. According to the Arrhenius equation:
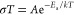
2where *A* is the pre-exponential factor determined by the structure, which is proportional to the concentration of mobile ions. In addition to the significant changes in activation energies as mentioned above, we further calculated the value of the pre-referential factor, which is expanded by 2 and 5 times from the bulk of sintered BZY to the surface of BZY and the interface of the heterostructure samples, indicating that the concentration of mobile ions is greatly increased on the surface and at the interface, which in turn leads to a great enhancement of the ionic conductivity from the bulk of sintered BZY (0.04 S cm^–1^) to the surface of BZY (0.09 S cm^–1^) and further the interface of the heterostructure CeO_2−δ_/BZY (0.23 S cm^–1^).

**Figure 4:**
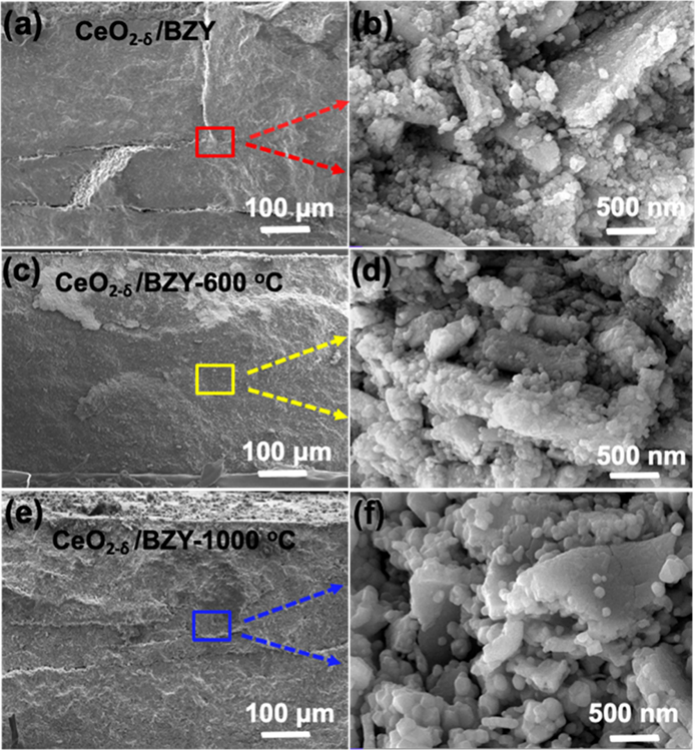
Cross-sectional SEM images of (a,b) CeO_2−δ_/BZY; (c,d) CeO_2−δ_/BZY-600 (sintered at 600 °C); and (e,f) CeO_2−δ_/BZY-1000 (sintered at 1000 °C).

**Figure 5:**
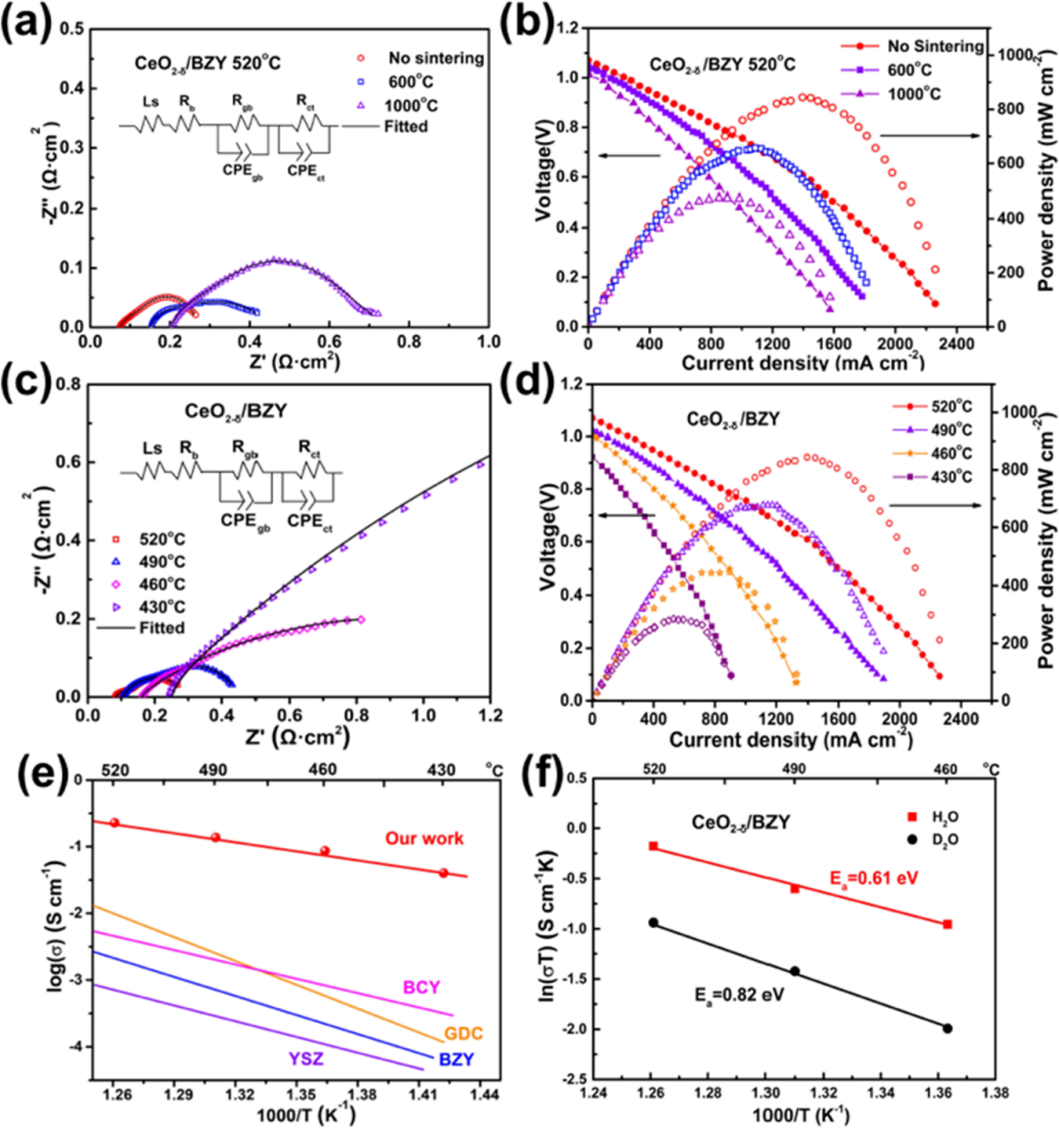
(a) Electrochemical impedance spectra of different CeO_2−δ_/BZY electrolytes prepared without and with sintering at different temperatures, and the data were obtained in H_2_/air fuel cell operation at 520 °C; (b) *I*–*V* and *I*–*P* curves obtained under the same conditions as in (a); (c) EIS of the CeO_2−δ_/BZY electrolyte tested at different operation temperatures, and (d) corresponding fuel cell *I*–*V* and *I*–*P* curves obtained under the conditions as in the (c). (e) Ionic conductivity of CeO_2−δ_/BZY compared with that of other oxygen ion-conducting electrolytes (dotted lines) and proton conductors (solid lines). BCY, barium-cerium/yttrium oxide; GDC, gadolinium-doped ceria; BZY, yttrium-doped barium zirconate; YSZ, yttrium-stabilized zirconia. (f) Arrhenius plots and calculated activation energies for ion conduction in the CeO_2−δ_/BZY electrolyte-based fuel cell device operated with D_2_O and H_2_O at various temperatures.

Furthermore, the CeO_2−δ_/BZY material was pressed into two pellets of 1.5 mm thickness and 1.3 mm in diameter, and one pellet was sintered at 600 °C and the other at 1000 °C, respectively. Figure 4 presents the cross-sectional SEM images for surface morphologies of the CeO_2−δ_/BZY sample before and after being subjected to the sintering at the two different temperatures. The average grain size of tens nanometer was observed with the CeO_2−δ_/BZY sample, and there exists a large contact interface between the particles, which may promote ion conduction and contribute to good electrochemical performance. After sintering, the diameter of nanosized particles increased, and the density increased from 6.25 g cm^–2^ of CeO_2−δ_/BZY to 6.66 g cm^–2^ of CeO_2−δ_/BZY-600 (sintered at 600 °C) and 7.25 g cm^–2^ of CeO_2−δ_/BZY-1000 (sintered at 1000 °C). CeO_2−δ_/BZY particles grew into a larger size with a greater flat face; the higher the sintering temperature, the larger the particle size and thus the greater the flat face as shown in Figure 4d,f. This may lead to a decrease of heterojunction interface with the increase of particle size and density when the calcination temperature was increased, e.g., from 600 to 1000 °C. Due to the loss of the surface and contact areas among particles, the interfacial conduction decreased with the increase of the sintering temperature, thus leading to the decreased conductivity (see EIS data in Figure 5a and fuel cell power output in Figure 5b).

Figure 5a displays EIS results of the heterostructure composites which were prepared with sintering at different temperatures of 600 and 1000 °C, respectively, as well as the one without sintering; the fuel cell was tested under a H_2_/air atmosphere at 520 °C in an open-circuit mode. The results can be simulated using the same equivalent circuit model as discussed above in Figure 2h. The CEP is the constant phase element representing a nonideal capacitor.^32^ The fitting values are shown in Table S2 in the Supporting Information. With the sintering, and with the increase of sintering temperature from 600 to 1000 °C, the resistance of the grain boundary, i.e., inter-hetero-surface, *R*_gb_, increased significantly from 4.14E–05 to 0.35 and 0.64 Ω cm^2^, suggesting that the ion conduction is hindered by minimizing the surfaces with the high-temperature sintering, thus the ionic conductivity at the interfaces is reduced.^33^ Correspondingly, the fuel cell power output decreased from 845 mW cm^–2^ (using the SIH electrolyte without sintering) to 657 mW cm^–2^ (using the SIH sintered at 600 °C) and further down to 476 mW cm^–2^ (using the SIH sintered at 1000 °C). These big differences in the power output further prove that the surface/interfacial conduction is much better than that of the bulk.

Figure 5c presents the EIS results of the CeO_2−δ_/BZY-based fuel cell running with H_2_/air at different testing temperatures. The resistance of the grain boundary increased from 4.14E–5 to 7.54E–2 Ω cm^2^ with decreasing testing temperatures from 520 to 430 °C, as shown in Table S3 (Supporting Information). Figure 5d displays the *I*–*V* and *I*–*P* curves of CeO_2−δ_/BZY fuel cell performance obtained at various temperatures. The maximum power densities of 845, 677, and 447 mW cm^–2^ were obtained at 520, 490, and 460 °C, respectively. The OCVs were all higher than 1.0 V. When the temperature decreased to 430 °C, the power output remained up to 286 mW cm^–2^. The corresponding ionic conductivity of CeO_2−δ_/BZY was measured in the fuel cell operation condition as presented in Figure 5e, and the conductivity of CeO_2−δ_/BZY obtained under fuel cell conditions exhibited a high value of 0.23 S cm^–1^at 520 °C. The conductivity of the CeO_2−δ_/BZY heterostructure sample is greatly enhanced compared to that of the BZY. In the intermediate operating temperature range (400 to 700 °C) for solid oxide fuel cells (SOFCs), this conductivity is significantly higher than the reported values from the state-of-art oxygen ions and proton-conducting electrolyte materials as seen in a comparison shown in Table 2.^34,35^

**Table 2:** Comparison of Proton Conductivity Values from Several Reported Electrolytes

materials	conductivity/S cm^–1^	*T*/°C
CeO_2−δ_/BZY (this work)	0.23	520
ZnO-LCP (La/Pr doped CeO_2_)^21^	0.156	550
CeO_2−δ_^36^	0.1	550
LixAl_0.5_Co_0.5_O_2_^37^	0.1	500
Ni_0.4_Zn_0.6_Fe_2_O_4_^15^	0.048	550
BaCe_0.7_Zr_0.1_Y_0.2_O_3−δ_^38^	2 × 10^–2^	600
Ba_3_Ca_1.18_Nb_1.82_O_9−δ_^39^	2 × 10^–3^	700

Furthermore, the isotopic effect was tested to provide evidence to confirm if the ion conduction is from protons.^40^ The diffusion of the deuteron would be slower than that of the proton if the H^+^ and D^+^ may both appear in CeO_2−δ_/BZY; this will lead to a marked conductivity difference in H_2_O or D_2_O vapor feed. According to classical theory, it can make a conductivity difference of , leading to a marked conductivity difference because of diffusion of the deuteron to be slower than that of the proton. According to the following equation:
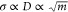
3where σ is the conductivity, *D* is the diffusivity, and *m* is the mass ratio of the diffusing species, e.g., *D_m_*/*H_m_* = 2. At various temperatures, as shown in Figure 5f, the associated conductivities were substantially decreased from H_2_O to D_2_O feed, and a higher activation energy was required, i.e., from 0.61 to 0.82 eV as shown in Figure 5f, which exhibits a clear H/D isotope effect and thus provides clear evidence of proton conduction being the case in the CeO_2−δ_/BZY heterostructure material.

**Figure 6:**
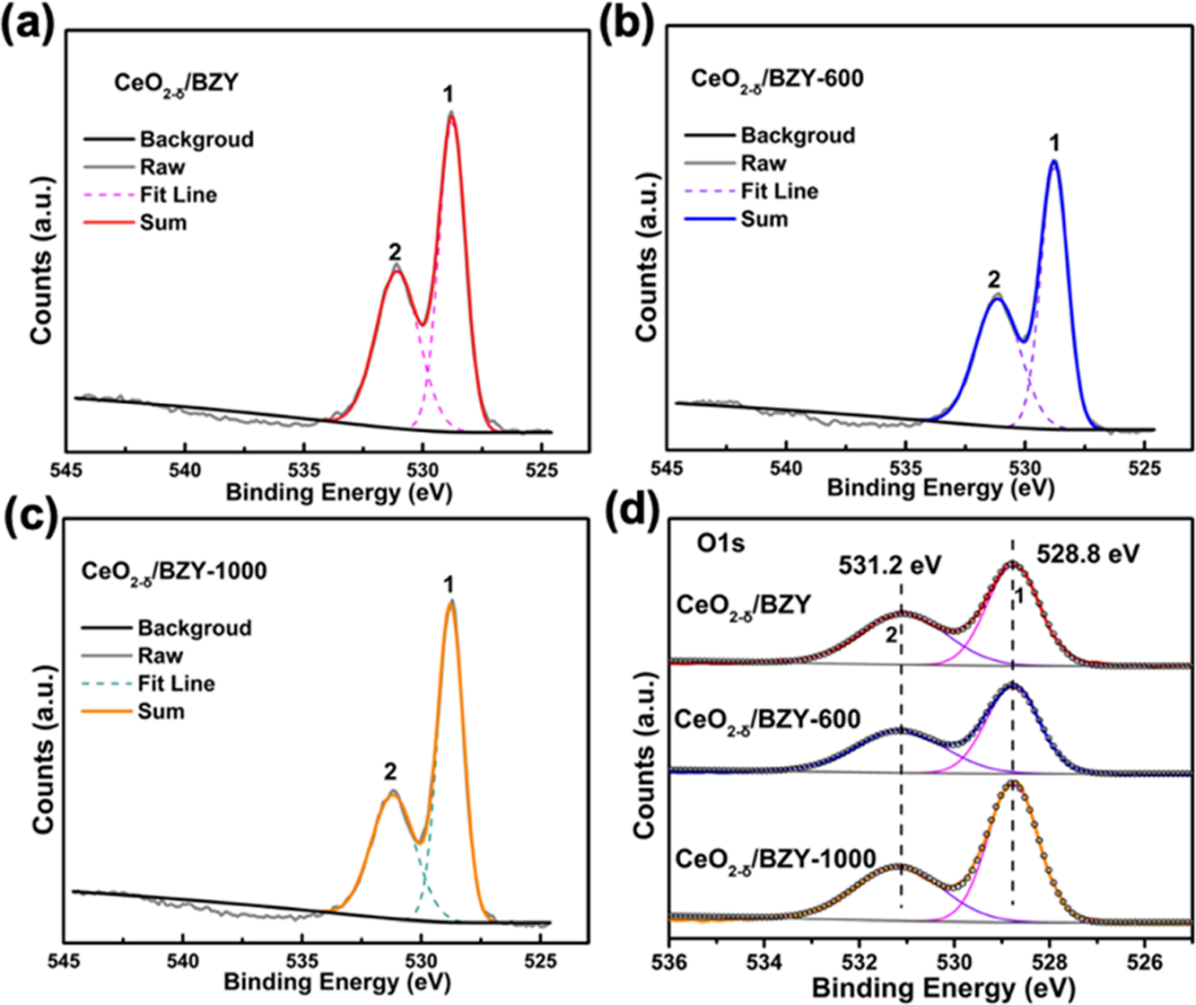
Fitting results for O 1s XPS spectra of (a) CeO_2−δ_/BZY, (b) CeO_2−δ_/BZY-600, and (c) CeO_2−δ_/BZY-1000; (d) comparison of the data obtained from (a–c) to see the effects of sintering at the two different temperatures of 600 and 1000 °C.

Figure 6 presents the XPS results in the O 1s region for CeO_2−δ_/BZY, without and with sintering at the two different temperatures of 600 and 1000 °C, respectively. The fitted background used is Shirley type. As shown in Figure 6a, the O 1s of CeO_2−δ_/BZY could be split into different symmetrical signals at 528.8 eV (marked as Peak 1) and 531.2 eV (marked as Peak 2). The signal at 528.8 eV is attributed to the lattice oxygen, and the peak at 531.2 eV corresponds to surface adsorbed oxygen.^21,28^ As listed in Table S4, with the high-temperature sintering, the ratio of O2/O1 decreased. It indicates that as the temperature increased, the grains grew up and the oxygen vacancy on the surface decreased due to the decrease of grain boundaries.^41^ Many previous studies reported that the oxygen vacancy is the main carrier of ion conduction.^42^ The decrease of oxygen vacancies hindered proton transport, which is consistent with the previous results of impedance and electrochemical performance tests.

**Figure 7:**
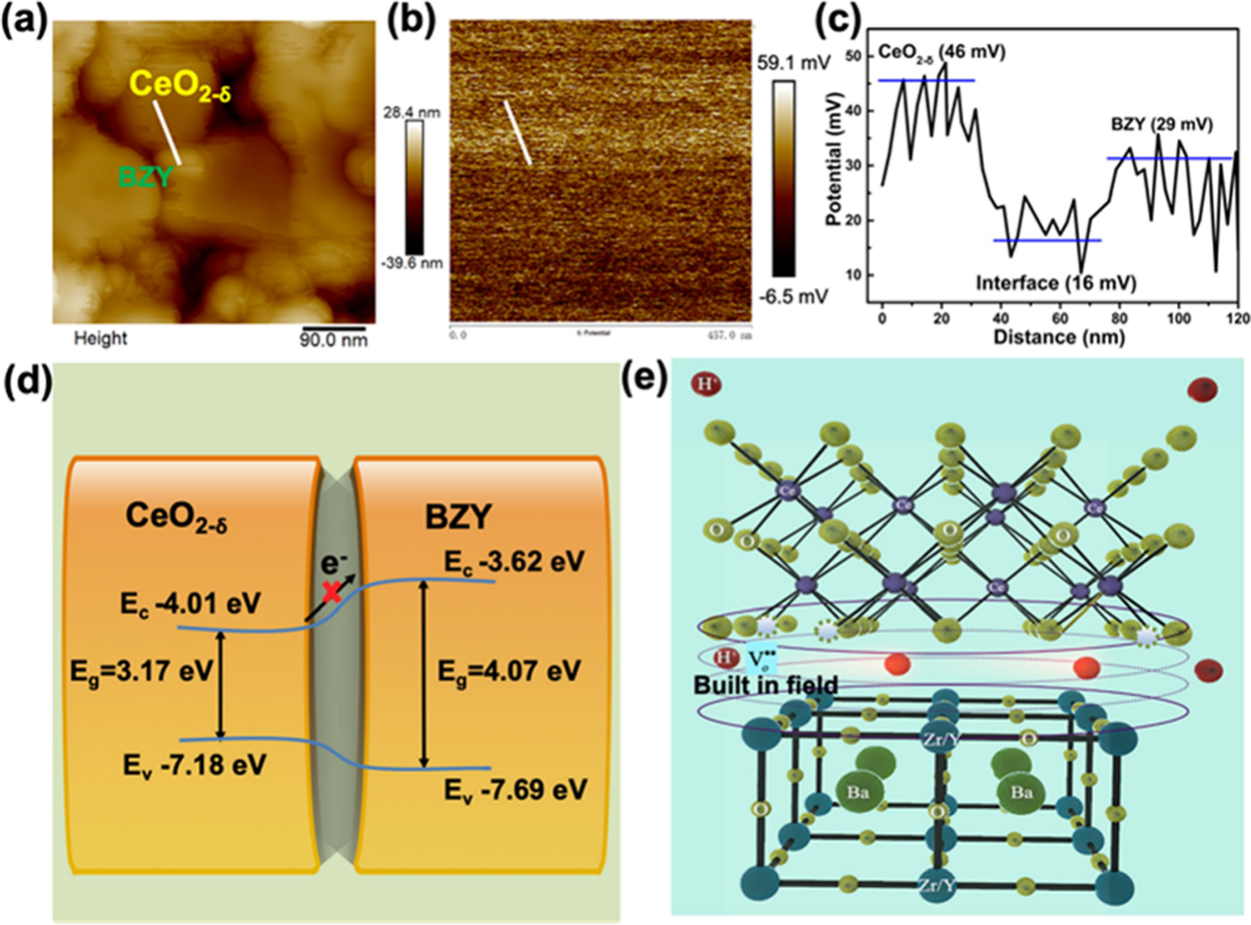
(a) Atomic force microscopy image and (b) scanning KPFM image of CeO_2−δ_/BZY. The scanning area is 2 μm by 2 μm. (c) Contact potential difference along the white line in (b). (d) Energy band mechanism diagram of CeO_2−δ_ and BZY. (e) Proton transport in the electrolyte membrane is constituted by the surface region of the CeO_2−δ_ and BZY.

KPFM was used to verify the existence of a BIEF at the CeO_2−δ_/BZY interface. Figure 7a,b shows the topographic images, while Figure 7c shows that the surface potential difference between CeO_2−δ_ and BZY is ∼17 mV, demonstrating the existence of a BIEF at the CeO_2−δ_/BZY interface. Considering the interface distance between two-phase particles being at less than nm level, see Figure 7c, such a potential (17 mV) corresponds to a strong interfacial electric field, *E* = *V*/*d*, where *E* is an LEF or BIEF at the interface, and *V* is the potential difference crossing CeO_2−δ_ and BZY particles; taking d at 1 nm (10^–9^ m) level, the *E* is 1.7 × 10^7^ V/m. Under such a strong BIEF, protons cannot stay static in the interface region, they must be highly mobile, or highly activated in motion. Therefore, proton transport is greatly enhanced for the CeO_2−δ_/BZY electrolyte by its heterostructures.

The UV–vis and UPS cutoff images of CeO_2−δ_ and BZY were taken as shown in Figure S3, and then the energy band mechanism diagram of CeO_2−δ_ and BZY is sketched and shown in Figure 7d. The bandgap (*E*_g_) of CeO_2−δ_ is 3.17 eV, and the *E*_c_ and *E*_v_ relative to the vacuum level are −4.01 and −7.18 eV, respectively. For BZY, the *E*_g_ is 4.07 eV, and the *E*_c_ and *E*_v_ are −3.62 and −7.69 eV, respectively. As CeO_2−δ_ and BZY have different energy levels, in order to reach the same Fermi level, the conduction band (CB) and valence band (VB) of the contact interface layer will be adjusted to form a barrier and BIEF. This case is also known as straddling alignment; the band gap value of the semiconductor CeO_2−δ_ is smaller than that of BZY. The potential of the VB of the semiconductor CeO_2−δ_ is located at a higher position while the CB is lower than that of BZY. In this scheme, electrons can only be transferred from CeO_2−δ_ to BZY, resulting in the accumulation of charge carriers in BZY;^43^ this facilitates, in turn, the recombination of the charge carriers and decreases the electron activity and mobility. Therefore, the electron cannot pass through the CeO_2−δ_/BZY heterostructure, which can then turn to prevent the electron from passing through the electrolyte (internal device), thus avoiding the short-circuiting problem. On the other hand, accumulated charges form a space charge zone resulting in the BIEF at the interface of the CeO_2−δ_/BZY heterostructure, which facilitates proton transport through the local electrical field across the interface.

The proton transport path in the CeO_2−δ_/BZY heterostructure is illustrated in Figure 7e. For CeO_2−δ_, the formation of oxygen defects is accompanied by the localization of Ce 4f state electrons and forms Ce^3+^ ions resulting in the existence of multiple oxidation states (Ce^4+^and Ce^3+^).^24^ The oxygen vacancy can promote the redox reaction of CeO_2−δ_ and the reconstruction of CeO_2_, and the formation process is described as the following formula 4. Protons can be transported through oxygen vacancies and oxygen defects caused by reduction to form (OH·) as described in formulas 5 and 6. The generated electrons are confined to the surface area due to the repulsion of positive charges, forming a BIEF. More importantly, the BIEF becomes a strong force to accelerate proton transport at the interfaces resulting in high interfacial proton conductivity and fuel cell power output.
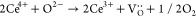
4
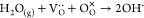
5
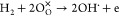
6

**Figure 8:**
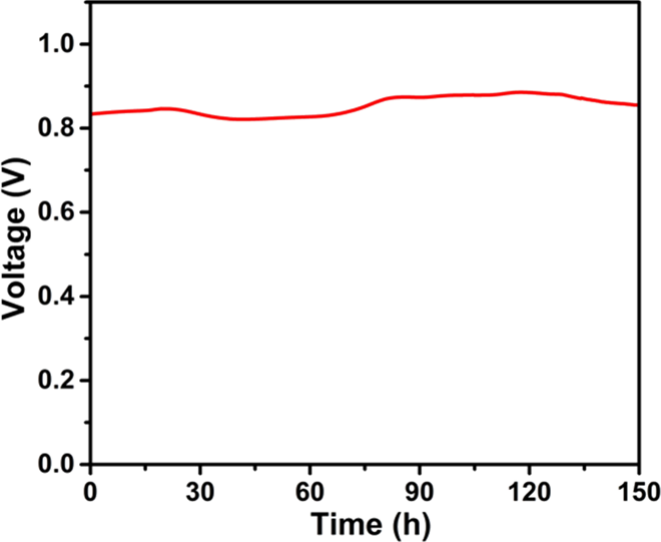
Stability test of the CeO_2−δ_/BZY electrolyte-based fuel cell operated at 520 °C under a constant current density of 100 mA cm^–2^.

A durability test of the CeO_2−δ_/BZY fuel cell was performed at 520 °C under H_2_/air conditions at a constant current density of 100 mA cm^–2^. The fuel cell exhibited a good stability during a period of 150 h as shown in Figure 8. The good fuel cell stability strongly confirms the functionality of the CeO_2−δ_/BZY electrolyte (SIH material) and the underpinning working principle. During the 28–40 h, there was a slight decrease in the cell voltage at a constant current density of 100 mA cm^–2^. This may be caused by the dynamic proton transport equilibrium process. Proton conduction is extrinsic in nature provided by the anode hydrogen oxidation reaction which produces protons that are continuously injected into the electrolyte. There is a balance situation or adjustment from the anodic injection (or electrode polarization) and proton transport sites at interfaces of the heterostructure electrolyte. Then the voltage began to increase after 40 h. This was due to the process of inserted proton concentrations being increased inside the SIH electrolyte, gradually tuning the CeO_2−δ_/BZY and reaching the highest proton conductivity. This is a unique phenomenon of external proton transportation.^6,44^ Based on the good stability tested over 150 h, the heterostructure provides a promising prospect for scaling up to meet practical applications. Furthermore, having the high-performance electrolyte, the fuel cell operation can be improved further by exploring optimized electrode materials.^44^

**Figure 9:**
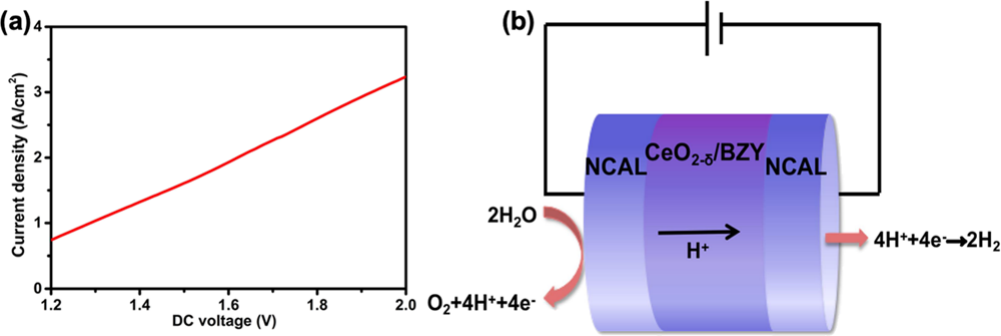
(a) Performance of the water electrolysis cell (reversed PCFC, current density as a function of the applied voltage) employing the CeO_2−δ_/BZY electrolyte at 520 °C. (b) Schematic of the reactions involved in the electrolysis cell with the water fed into the positive electrode.

The SOFC converts the chemical energy of a fuel (H_2_) into electrical energy output with clean water as a byproduct. It can also be operated reversely, as an electrolytic cell (named solid oxide electrolysis cell, SOEC), to use electricity to split water into hydrogen and oxygen gases, i.e., converting electrical energy into chemical energy in H_2_. A similar proton transport mechanism in the electrolyte holds for the SOEC.^45,46^ The driving forces for the proton transport through the internal electrolyte layer depend on the potential difference (voltage applied) across the cell and the concentration difference at the two ends of the electrolyte (anode and cathode sides). In the voltage scanning range of 0∼2 V, the positive electrode was fed with water vapor, the current was recorded as a function of the voltage applied, and the water electrolysis polarization curve of the CeO_2−δ_/BZY heterostructure-based cell was recorded and is shown in Figure 9a. The current density generated by the electrolysis cell reached 3.2 A cm^–2^ at 2.0 V, which is higher than the recently reported data.^47,48^ This high current density provides further evidence that the CeO_2−δ_/BZY electrolyte possesses a high proton conductivity. A schematic of the reactions and proton transport involved in the water electrolysis cell is shown in Figure 9b, where the water (H_2_O) on the positive electrode (now the anode side in the electrolysis mode) is oxidized to O_2_, releasing protons (H^+^) and electrons (e^–^); the protons are transported to the negative electrode (now cathode side) through the internal electrolyte while electrons are transmitted to the negative electrode via the external circuit; hydrogen (H_2_) is formed at the negative electrode (cathode) by the reduction reaction of H^+^ with electrons.^49^ Faraday efficiency is 100% for the hydrogen evolution reaction (two-electron process) at the cathode and oxygen evolution reaction (four-electron process) at the anode, and the current is only from the electrolysis of H_2_O. The volume ratio of H_2_ and O_2_, collected at the cathode and anode, respectively, is 2:1. Thus the molar ratio of H_2_:O_2_ from the electrolysis of H_2_O is 2:1. Our studies illustrate that the CeO_2−δ_/BZY heterostructure composite can be employed as a high proton conducting electrolyte for both fuel cell and electrolysis operations, with great potential for highly efficient green hydrogen production and wider applications including electrosynthesis as well as various fuel cells.

## 4 Conclusions

By tuning the BaZr_0.8_Y_0.2_O_3_ (BZY) low bulk conduction to CeO_2−δ_/BZY SIH high interfacial conduction, the SIH was developed as a novel proton conducting material with a record-high proton conductivity of 0.23 S cm^–1^ at 520 °C that is much higher than 0.10 S cm^–1^ threshold which is also offered by the state-of-the-art commercial Nafion membrane. The PCFC employing this SIH as the electrolyte exhibited a high power density of 845 mW cm^–2^ at 520 °C, which is much higher than 229 mW cm^–2^ obtained from the PCFC using BZY as the electrolyte under the same conditions. A high limited current density of 2.3 A cm^–2^ was achieved for the SIH-based fuel cell at 520 °C. Furthermore, under the reversed fuel cell operation, i.e., water electrolysis, a very high current density of 3.2 A cm^–2^ was achieved at an applied voltage of 2.0 V at 520 °C for the SIH-based water electrolysis cell. These results provide solid evidence that the SIH material CeO_2−δ_/BZY possesses a superb proton conductivity.

The interfacial conduction was identified first through an observation of the grain boundary conduction of BZY to be significantly higher than that of the bulk. The high-temperature sintering approach reveals a low bulk proton conduction of the sintered BZY, and then a higher surface (grain boundary) proton conduction of BZY without high-temperature treatment was identified. The latter inspired us to further develop the SIH material CeO_2−δ_/BZY which possesses rich interfaces and thus exhibits a superb proton transport property. Tuning the band structures generates a BIEF, which could accelerate the proton transport leading to a high ionic conduction. This work provides a deep understanding of the proton transport mechanism over interfaces of the semiconductor–ionic heterostructure that is far beyond the bulk, leading to a new strategy to design high interfacial proton conduction to facilitate the recent development of advanced proton conducting ceramic electrolytes. This new methodology is remarkably different from, and far more efficient than, that of the traditional structural doping within bulk conduction. This work demonstrates a highly efficient new strategy by tuning the low bulk to high interfacial proton-conducting to meet the emerging demands of the sufficient proton conductivity for advanced electrochemical energy technologies, e.g., fuel cells and water electrolysis for green hydrogen production and wider applications.

## Supporting Information Available

SEM image and EDS elemental mapping images of the CeO_2−δ_/BZY heterostructure and *I*–*V* curves, XPS, UV spectra, UPS plots, and EIS fitted data of various samples (PDF)
