# Supplementary material for: Designing High Interfacial Conduction beyond Bulk via Engineering the Semiconductor–Ionic Heterostructure CeO2−δ/BaZr0.8Y0.2O3 for Superior Proton Conductive Fuel Cell and Water Electrolysis Applications
Source: ACS Appl Energy Mater. 2022 Dec 15;5(12):15373–84. doi: 10.1021/acsaem.2c02995 (PMC9795487; doi:10.1021/acsaem.2c02995)
Supplement: Supplementary file 1 — ae2c02995_si_001.pdf [file ae2c02995_si_001.pdf]

Supplementary Information

Designing high interfacial conduction beyond bulk via engineering semiconductor-ionic heterostructure $CeO_{2-\delta}/BaZr_{0.8}Y_{0.2}O_3$ for superior proton conductive fuel cell and water electrolysis applications

Yueming Xing[a], Bin Zhu[,a, b], Liang Hong[c], Chen Xia[d], Baoyuan Wang[d], Yan Wu[a], Hongdong Cai[d], Sajid Rauf[e], Jianbing Huang[f], Muhammad Imran Asghar[d, g], Yang Yang[c], and Wen-Feng Lin[*,c]*

[a]*Engineering Research Center of Nano-Geo Materials of Ministry of Education, Faculty of Materials Science and Chemistry, China University of Geosciences, No. 388 Lumo Road, Wuhan 430074, China*

[b]*Jiangsu Provincial Key Laboratory of Solar Energy Science and Technology/ Energy Storage Joint Research Center, School of Energy & Environment, Southeast University, Nanjing, 210096, China.*

[c]*Department of Chemical Engineering, Loughborough University, Loughborough, Leicestershire LE11 3TU, United Kingdom. Email: w.lin@lboro.ac.uk*

[d] *Hubei Collaborative Innovation Center for Advanced Organic Materials, Faculty of Physics and Electronic Science, Hubei University, Wuhan 430062, China*

[e] *College of Electronics and Information Engineering, Shenzhen University, Guangdong Province, 518000, China*

[f] *State Key Laboratory of Multiphase Flow in Power Engineering, Xi'an Jiaotong University, Xi'an, 710049, China*

[g] *New Energy Technologies Group, Department of Applied Physics, Aalto University School of Science, P. O. Box 15100, FI-00076 Aalto, Espoo, Finland*

*** Corresponding Authors**

E-mails: w.lin@lboro.ac.uk (W.F. Lin); zhu-bin@seu.edu.cn (B. Zhu).

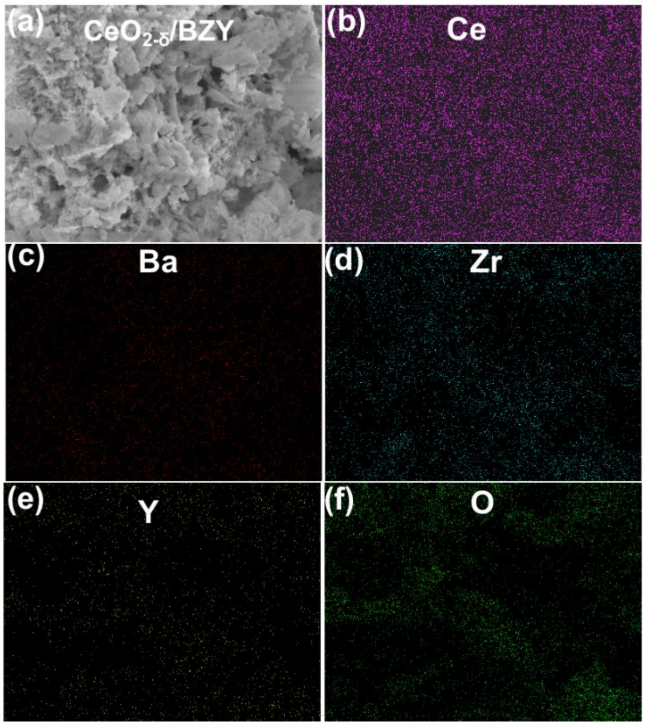

Figure S1. (a) SEM image and (b-f) EDS elemental mapping images of $CeO_{2-\delta}$/BZY heterostructure.

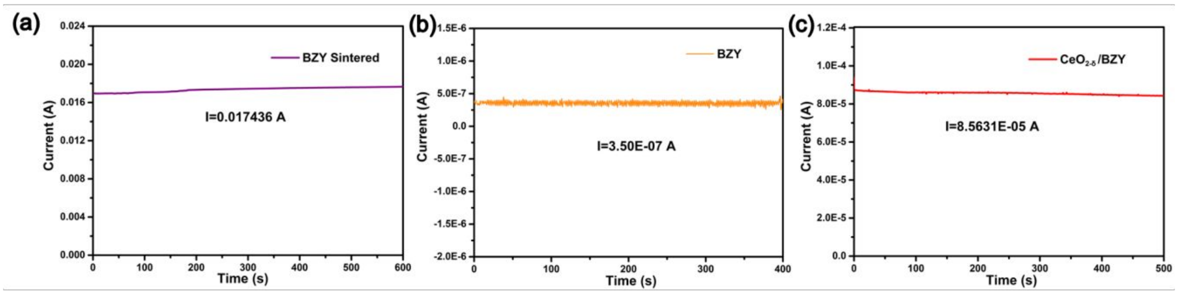

Figure S2. *I-V* curves of (a) BZY, (b) BZY Sintered and (c) $CeO_{2-\delta}$/BZY obtained from DC method tested with a bias voltage of 1.0 V.

Table S1. EIS fitted data of the BZY and BZY-Sintered composites, measured at 520 ^{o}C.

	Ls	R_b/ Ω	R_{gb}/ Ω	R_{ct}/ Ω
BZY	9.00E-08	0.40	0.39	1.15
BZY-Sintered	1.05E-08	0.65	2.95	4.19

Table S2. EIS fitted data of the $CeO_{2-\delta}$/BZY composite without sintering and with sintering at the two different temperatures of 600 and 1000 ^{o}C, measured at 520 ^{o}C.

T	Ls	R_b	R_{gb}	Q_1	n	C	R_{ct}	Q_2	n
$CeO_{2-\delta}$/BZY	6.82E-08	0.083	4.14E-05	1.58E-08	1.040	4.65E-08	2.72E-01	2.440	0.414
$CeO_{2-\delta}$/BZY-600	1.30E-07	0.125	3.48E-01	2.36E-02	0.312	5.94E-07	1.90E+00	3.280	0.116
$CeO_{2-\delta}$/BZY-1000	1.12E-07	0.195	6.39E-01	2.05E-03	0.503	2.91E-06	5.99E-01	0.026	0.542

Table S3. EIS fitted data of the $CeO_{2-\delta}$/BZY composite obtained under four different testing temperatures.

T	Ls	R_b	R_{gb}	Q_1	n	C	R_{ct}	Q_2	n
520°C	6.82E-08	0.083	4.14E-05	1.58E-08	1.040	4.65E-08	2.72E-01	2.440	0.414
490 ^{o}C	4.93E-08	0.102	2.28E-04	1.37E-05	0.821	1.70E-07	3.63E-01	0.739	0.458
460 ^{o}C	7.57E-08	0.142	3.54E-03	1.13E-03	0.599	1.28E-06	1.43E+00	0.949	0.344
430 ^{o}C	6.89E-08	0.199	7.54E-02	1.42E-03	0.628	6.33E-06	5.80E+00	0.059	0.429

Table S4. The peak areas at different positions of O1s peak of the $CeO_{2-\delta}$/BZY composite without sintering and with sintering at the two different temperatures of 600 and 1000 ^{o}C.

Peak	Position BE (eV)	FWHM (eV)	Raw area (cps. eV)	Atomic Conc
$CeO_{2-\delta}$/BZY				
O1	528.76	1.32	40198.55	54.7%
O2	531.07	2.14	33224.84	45.3%
Area O2/O1	/	/	/	**0.83**
$CeO_{2-\delta}$/BZY-600				
O1	528.78	1.34	35255.23	56.1%
O2	531.14	2.22	27537.75	43.8%
Area O2/O1	/	/	/	**0.78**
$CeO_{2-\delta}$/BZY-1000				
O1	528.77	1.27	53032.04805	59.8%
O2	531.18	2.17	35629.62077	40.1%
Area O2/O1	/	/	/	**0.67**

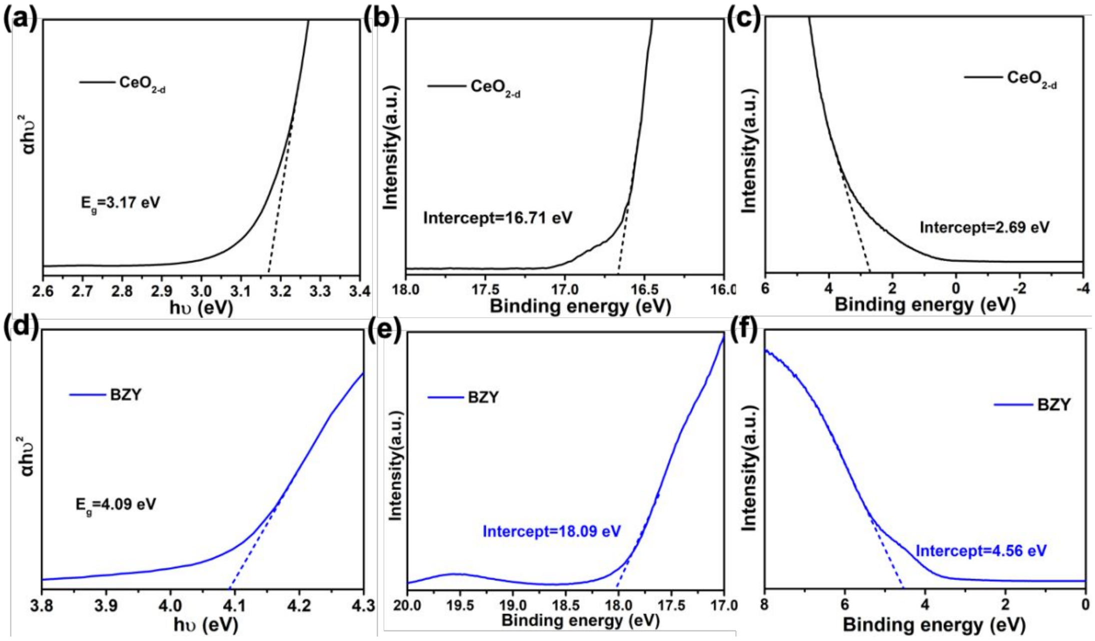

Figure S3. (a) UV spectrum and (b-c) UPS plots with the magnified views of the low binding energy cutoff of $CeO_{2-\delta}$; (d) UV spectrum and (e-f) UPS plots with the magnified views of the low binding energy cutoff of BZY.
